# MiR-489-3p Reduced Pancreatic Cancer Proliferation and Metastasis By Targeting PKM2 and LDHA Involving Glycolysis

**DOI:** 10.3389/fonc.2021.651535

**Published:** 2021-11-12

**Authors:** Dan Zhang, Zhiwei He, Yiyi Shen, Jie Wang, Tao Liu, Jianxin Jiang

**Affiliations:** ^1^ Department of Hepatobiliary Surgery, Renmin Hospital of Wuhan University, Wuhan, China; ^2^ Department of Hepatic-Biliary-Pancreatic Surgery, The Affiliated Hospital of Guizhou Medical University, Guiyang, China

**Keywords:** miR-489-3p, proliferation, glycolysis, pancreatic cancer, metastasis

## Abstract

**Introduction:**

Malignant proliferation and metastasis are some of the causes of high mortality in pancreatic cancer. MicroRNAs have been a hot spot in cancer research and are involved in tumor formation and metabolic stress responses. However, the biology function and underlying mechanism of miRNA regulating pancreatic cancer progress is remained uncleared.

**Methods:**

RNA-seq analysis the glycolysis associated miRNAs and verified miRNA-489-3p was involving in glycolysis. We used RNA *in situ* hybridization (ISH) and qRT-PCR to analyze the differential expression of miR-489-3p in pancreatic cancer tissues and adjacent tissues and cell lines. Then the function assay of *in vivo* and *in vitro* were used to evaluated the role of miR-489-3p in the proliferation, metastasis and glucose metabolism of pancreatic cancer. Furthermore, dual luciferase reporter and rescue experiments were performed to explore the mechanism underlying in the role of miRNA-489-3p.

**Results:**

We determined that glycolysis associated miRNA miR-489-3p was downregulated in pancreatic cancer tissues and cell lines. The gain and loos of function experiments confirmed that miR-489-3p could inhibit the proliferation, metastasis and glucose metabolism of pancreatic cancer. Further, we found that miR-489-3p could target regulating LDHA and PKM through the luciferase report experiment. Finally, *in vivo* experiment confirmed that highly expressed miR-489-3p inhibited the growth of pancreatic cancer.

**Conclusion:**

In short, this study identified miR-489-3p as a novel therapy target for pancreatic cancer which was involving in the proliferation, metastasis and glycolysis, but its diagnostic value deserves further study.

## Introduction

Pancreatic ductal adenocarcinoma (PDAC) is a lethal cancer with poor treatment methods available (1). Early metastasis and invasion are the main factors leading to its poor prognosis, only sufficient energy and biosynthetic precursors can sustain this aggressive biology (2, 3). However, one of the characteristics of the pancreatic cancer microenvironment is the proliferation of dense connective tissue, which leads to increased pressure inside the tumor and compresses the vascular system (4, 5). Therefore, its hypovascularization decreases the supply of materials for biosynthesis into cancer cells and generates an energy shortage. Nevertheless, tumor cells can adapt to this change by metabolic reprogramming; the most typical example of this is enhanced glycolysis, which was initially named as the “Warburg Effect” (6). This reprogramming provides energy, macromolecular precursors which are crucial to the abnormal growth and survival of cancer cells (7). Although aerobic glycolysis has been verified in PDAC, its driving mechanism remains hardly known. Hence, the elucidation of this mechanism is essential for the research and treatment of pancreatic cancer.

MicroRNAs, a group of non-coding RNAs of 18−23 nucleotide, are high-profile molecular family that participates in mediating metabolic stress response in cancer (8–10). For instance, they are widely involved in the regulation of signal pathways such as p53, LKB1/AMPK, c-Myc, and other pathways that regulate metabolic response (11–13). It has been reported that microRNAs can, directly and indirectly, promote glycolysis of a variety of tumors, such as pancreatic cancer (14), gastric cancer (15), bladder cancer (16). Furthermore, literature has found multiple micro RNAs including miR-135 (14) and miR-124 (17) to be implicated in the metabolic reprogramming of pancreatic cancer. Since miR-489-3p has been reported to inhibit tumor progression (18, 19), we hypothesized that it can also inhibit the progression of pancreatic cancer and that it functions against glycolysis that is known to promote tumor progression. In the tumor disease deterioration progress, the glycolysis metabolic enzyme exerted vital role in the cancer cell viability and distant metastasis, through producing more nucleic acid raw materials or protecting cells from oxidative damage (20). Pyruvate kinases catalyzes the transfer of a phosphoryl group from phosphoenolpyruvate to ADP, generating ATP and pyruvate in the tumor progress (21). Meanwhile, the glycolysis enzymes could influence HIF-1A transcriptional activity that regulating tumor cell viability in the hypoxia environmental (22).

In this study, we found that miR-489-3p was downregulated and involved in glucose metabolism reprogramming and malignancy in pancreatic cancer (PC). To further explore the function of the miR-489-3p, we conducted loss- and gain-of-function assays to observe the role of miR-489-3p in proliferation and metastasis *in vivo* and *in vitro*, as well as glucose metabolism in PC. The results proved that miR-489-3p inhibits malignancy and glucose metabolism and targets the critical enzymes for glycolytic flux, LDHA, and PKM2, thus controlling glycolysis and PC progression.

## Materials and Methods

### Human Pancreatic Tumor Samples

A total of 90 cases of pancreatic cancer tissues and adjacent tissues from the pancreatic surgery department of Wuhan University People’s Hospital from 2009 to 2019 were collected for RNA *in situ* hybridization. All patients were diagnosed with pancreatic cancer according to the World Health Organization’s diagnostic criteria. All samples were approved by the ethics review committee and the patient’s informed consent was obtained.

### Cell Culture

The pancreatic epithelial cell lines HPDE and PC (AsPc-1, BXPC-3, Capan-2, CFPAC-1, PANC-1, MIA PaCa-2, and SW1990) were obtained from the American Type Culture Collection. HPDE, AsPc-1, and BXPC-3 cells were maintained in RPMI-1640 medium (Gibco) containing 10% fetal bovine serum (FBS; Gibco), while Capan-2, CFPAC-1, PANC-1, MIA PaCa-2, and SW1990 were cultured in DMEM containing 10% FBS. Both cell lines were cultured at 37°C in a humidified 5% CO2 incubator according to ATCC protocols.

### *In Situ* Hybridization

The expression of miR-489-3p in PC and adjacent normal tissues was analyzed using biotin-labeled miR-489-3p probes (Guangzhou RiboBio Co., Ltd.). Paraffinized PC tissues were deparaffinized with different concentrations of xylene and ethanol. The PC tissues were then incubated with miR-489-3p probes overnight at 40°C. Digoxin substrate was used to visualize miR-489-3p signals, and cell nuclei were stained with hematoxylin.

### Cell Viability Analysis

CCK8 (Cell Counting Kit-8, Beyotime, China) was used to detect the proliferation of PC cells. Following the instructions of the manufacturer, 100ul of 2x10^3^ cells were seeded into a 96-well plate, and 10ul of CCK8 reagent was added to each well. Absorbance values were measured every 0, 24, 48, 72, and 96 hours and recorded for statistical analysis.

### Colony Formation Assay

The PC cells were seeded into six-well plates at a concentration of 500 cells per well and cultured at 37°C and 5% CO2 for two weeks. Then, the cultures were fixed with 4% paraformaldehyde for 20 minutes and followed by staining with 0.2% crystal violet for 30 minutes. The number of clones was observed under an optical microscope and analyzed with statistical software.

### Transwell Assays

The PC cells of different treatment groups were seeded into the upper chamber of Transwell in a concentration of 1x10^5^ per well. 200 ul of a medium containing 10% serum was added to the upper chamber, and 700 ul of serum-free medium added to the lower chamber. The transwell was then incubated for 24 hours at 37°C after which the culture medium was discarded. The transwell was washed with PBS (Hyclone, USA), and fixed with 4% paraformaldehyde (Biosharp, China) for 30 minutes. It was then dyed using 0.5% crystal violet (Solarbio, Beijing, China) solution for 30 minutes. Finally, the transwell was rinsed with pure water, observed under an optical microscope, and images taken.

### Quantitative Real-Time PCR

The cells of each group were seeded in a six-well plate, and the total RNA of each group was extracted using Trizol reagent (Invitrogen, CA, USA) when the cells achieved 80% - 90% confluence. The cDNA was then synthesized using a PrimeScript RT reagent kit (Takara) according to the manufacturer’s instructions. Real-time quantitative PCR was performed by Powerup SYBR Green PCR Master Mix (Life Technologies). The primer sequences used in the present study were as follows:

miR-489-3p Forward primer (5′ to 3′): AGGGGGTGACATCACATATAC

miR-489-3p Reverse primer (5′ to 3′): GAGAGGAGAGGAAGAGGGAA

U6 Forward primer (5′ to 3′): CTCGCTTCGGCAGCACA

U6 Reverse primer (5′ to 3′): AACGCTTCACGAATTTGCGT

GAPDH Forward primer (5′ to 3′): CTCCAAAATCAAGTGGGGCG

GAPDH Reverse primer (5′ to 3′): TGGTTCACACCCATGACGAA

LDHA Forward primer (5′ to 3′): ATGGCAACTCTAAAGGATCAGC

LDHA Reverse primer (5′ to 3′): CCAACCCCAACAACTGTAATCT

PKM2 Forward primer (5′ to 3′): ATGTCGAAGCCCCATAGTGAA

PKM2 Reverse primer (5′ to 3′): TGGGTGGTGAATCAATGTCCA

### Luciferase Reporter Assay

The cells were seeded into a six-well plate. and transfected using lipofectamine reagent (Invitrogen) with miR-489-3p or luciferin Enzyme reporter genes for 4-6 hours. The medium was replaced with DMEM and then cultured in an incubator at 37°C for 24 hours. Then disposed according to the product instructions for the double luciferase reporter gene detection kit (Beyotime Biotechnology, China). Briefly, 500 microliters of reporter gene cell lysate were discarded after discarding the medium, and the supernatant was taken for determination.

### Western Blot

The cells of each group were seeded in a six-well plate, and the protein was obtained by lysis with RIPA buffer (beytime, China). BCA reagent (beytime, China) was used to quantify the protein concentration according to the Standard protein, and then the protein was diluted to the same concentration, and boiled for 5 minutes. Electrophoresis was performed using Solarbio reagents and 10% separation gel and 5% concentrated gel were prepared according to the manufacturer’s instructions. After electrophoresis and transfer, bands were then visualized using ECL reagent (Boster Biological Technology Co. Ltd.) with a chemiluminescence imaging system (Bio-Rad). The primary antibodies used in the present study were as follows: GAPDH (60004-1-Ig, Proteintech), PKM2(15822-1-AP, Proteintech), LDHA (21799-1-AP, Proteintech).

### Glucose Uptake and Lactate Production Measurements

Glucose uptake was determined using the D-2-deoxyglucose method following the manufacturer’s instructions. D-2-deoxyglucose used was purchased from Beyotime, China. Lactic acid production was evaluated using a Lactic Acid detection kit (Leagene Biological, Beijing) as the recommendations of the manufacturer.

### Cellular ATP Level

Experiments were performed using an ATP detection kit (Beyotime Biotechnology, China). Add 200 μl of lysate to each well of the six-well plate according to the product instructions, and centrifuge the supernatant after full lysis. Prepare ATP working solution and add it to the detection tube to measure the luminescence value.

### Extracellular Acidification Rate

Hippocampal experiments were performed using Agilent equipment: Hippocampus XFe24 Micro Edition and XFe24 cartridge. Subsequently, cells (1×10^4^ cells/well) were seeded into 96-well XF cell culture microplates in selective medium. 56 μl glucose (100 mM), 62 μl oligomycin (10 μM) and 69 μl 2DG (50 mM) were added to the cartridge wells. The ECAR values were measured and recorded for statistical analysis.

### *In Vivo* Assay

A total of 10^7^ PANC-1 cells (miR-NC group and miR-489-3p up-regulation group) were injected subcutaneously into 4 weeks old female NCr nude mice (Hua Fukang Biotechnology, Beijing) for tumor growth. The tumor size was measured with calipers every 7 days for a period of 35 days. At the end of the experiment, the mice were euthanized, and subcutaneous tumors were collected for further analyses.

### IHC Analysis

Immunohistochemical staining was used to detect the expression of proliferation and metabolic indicators. Briefly, the subcutaneous tumor tissue was cut into 3um sections and then dewaxed. The sections were then incubated with the primary antibodies KI-67(27309-1-AP, Proteintech), PCNA (10205-2-AP, Proteintech), GLUT1 (21829-1-AP, Proteintech), PKM2(15822-1-AP, Proteintech), LDHA (21799-1-AP, Proteintech), HK2(66974-1-Ig, Proteintech) at 4°C overnight. After washing three times with PBS, each piece was stained with 3,3 ‘diaminobenzidine (DAB) and then observed and photographed under the light microscope (Nikon, Tokyo, Japan).

### Statistical Analyses

The results were analyzed and presented as the mean ± standard deviation. GraphPad Prism 7.0 (San Diego, California, USA) was used for mapping and statistical analysis. Chi-square test was used to analyze the relationship between miR-489-3p expression level and clinicopathological characteristics in PC. The Kaplan-Meier curve method was used to analyze the overall survival rate. The student’s t-test was used for statistical comparison between the two groups. Significance level was set at P <0.05 (*) and P <0.05 (**).

## Results

### The Expression of miR-489-3p and Its Relationship With Clinical Prognosis of Patients

**Figure 1:**
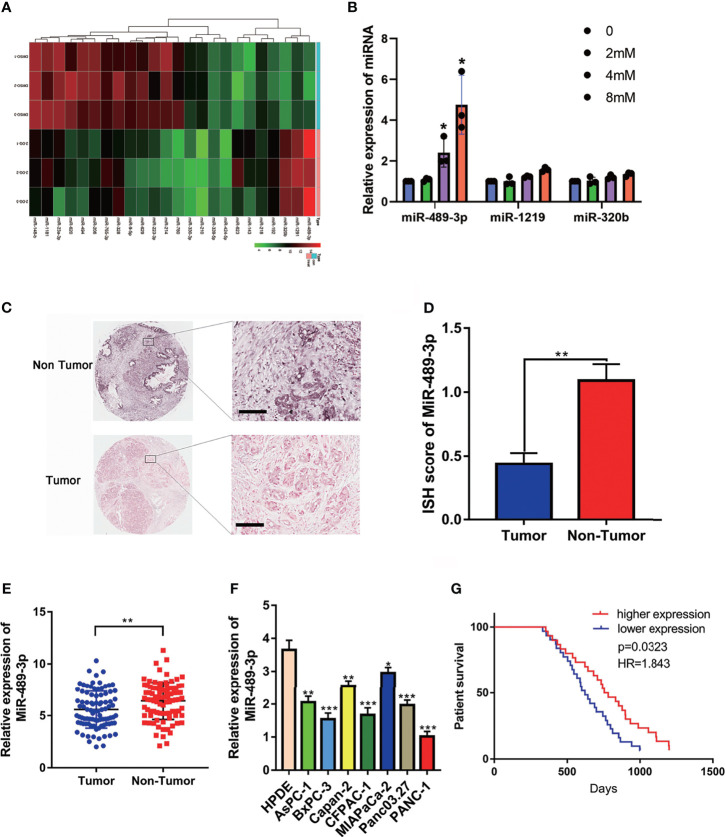
The expression of miR-489-3p and its relationship with clinical prognosis of patients. **(A)** RNA-seq analysis the different expressed miRNAs associated with glycolysis. **(B)** q-RT-PCR analysis the upregulated miRNA in the exposure with glycolysis inhibitor 2-DG. **(C, D)** RNA *in situ* hybridization experiments showed the expression of miR-489-3p in paracancerous and cancerous tissues. The bar stands for 50 microns **(E)** qRT-PCR analysis of the relative expression of miR-489-3p in adjacent tissues and PC tissues. **(F)** qRT-PCR showed the relative expression of miR-489-3p in PC cell lines and pancreatic normal duct epithelial cells (HPDE). **(G)** Kaplan-Meier curve was divided into survival periods by miR-489-3p expression. Among them, patients were divided into high expression group (red) and low expression group (blue) by median expression of miR-489-3p. (*P < 0.05, **P < 0.01, ***P < 0.001).

To explore the potential miRNAs involving in the glycolysis, we detected the different expressed miRNAs by RNA-seq. Then we verified the upregulated miRNAs by q-RT-PCR, we found that miR-489 was upregulated in the PANC-1 cell which exposed with 2-DG, an inhibitor of glycolysis (**Figures 1A, B**). RNA *in situ* hybridization (ISH) experiment was performed on the tumor tissues of 90 patients with pancreatic cancer to verify the expression of miR-489-3p. We found that miR-489-3p expression in tumor tissues was much lower than in the adjacent tissues (**Figures 1C, D**). This finding was confirmed by the qRT-PCR analysis in the PC tissues and cell lines (**Figures 1E, F**). Based on the pathological characteristics and miR-489-3p expression of pancreatic cancer (**Table 1**), we found that miR-489-3p expression was significantly related to tumor size (P < 0.01) and distant metastatic ability (P < 0.05). When we performed survival analysis in differently miRNA expression patients, it was found that patients with low expression of miR-489-3p had a worse prognosis (P=0.0323, HR=1.843; **Figure 1G**).

**Table 1:** Association of miR-489-3p expression with clinicopathological features from PC patients.

Clinical Epidemiology and Clinicopathologic Feature		miR-489-3p		*p* value
		low expression	high expression	
**all cases**		**41**	**49**	
**age**				
**≤50**		**11**	**6**	**0.0783**
**＞50**		**30**	**43**	
**gender**				
**male**		**21**	**29**	**0.4489**
**female**		**20**	**20**	
**diameter of tumor**				
**≤3**		**7**	**18**	**0.0381**
**＞3**		**34**	**31**	
**pathological grading**				
**Ⅰ**		**2**	**4**	**0.1366**
**Ⅱ**		**32**	**30**	
**Ⅲ**		**7**	**15**	
**Ⅳ**		**0**	**0**	
**lymphatic metastasis**				
**N0**		**30**	**41**	**0.2240**
**NI**		**11**	**8**	
**distant metastasis**				
**M0**		**34**	**48**	**0.0126**
**M1**		**7**	**1**	
**AJCC clinical staging**				
**Ⅰ**		**6**	**16**	**0.0072**
**Ⅱ**		**10**	**17**	
**Ⅲ**		**24**	**15**	
**Ⅳ**		**1**	**1**	

### MiR-489-3p Inhibits Proliferation and Invasion of Pancreatic Cancer

**Figure 2:**
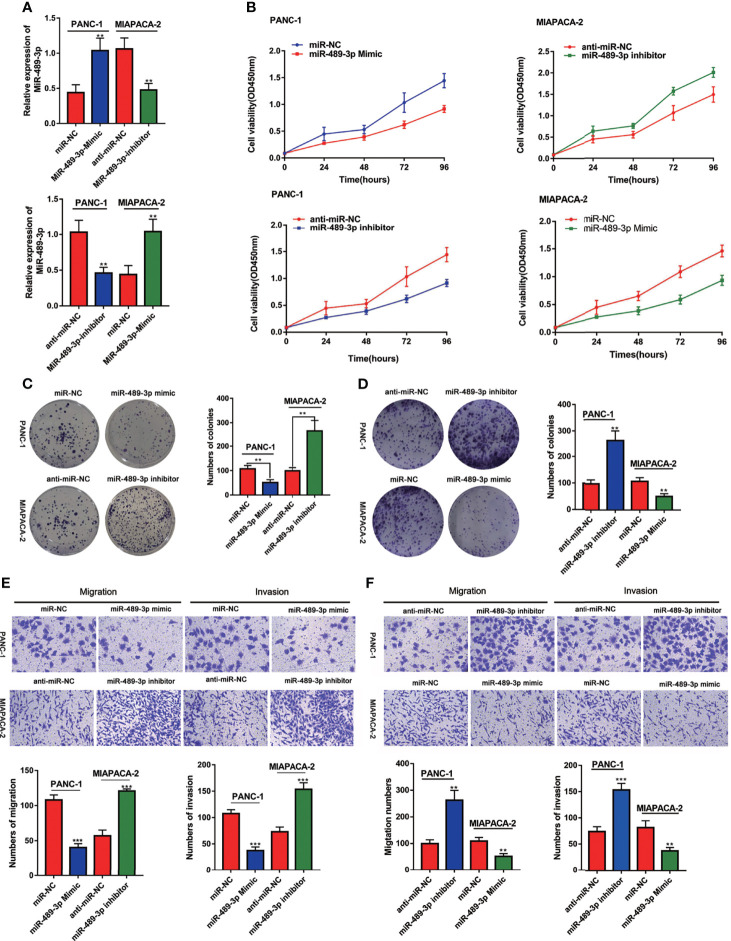
MiR-489-3p inhibits proliferation and invasion of pancreatic cancer. **(A)** qRT-PCR showed the relative expression of miR-489-3p after transfection of miR-489-3p mimic and inhibitor in PC cells. **(B–D)** CCK8 and plate cloning assays showed the cell proliferation ability of PC cells transfected with miR-489-3p mimics and miR-489-3p inhibitors. **(E, F)** Transwell migration and invasion assays showed the cell migration and invasion ability of PC cells transfected with miR-489-3p mimics and inhibitors. (**P < 0.01, ***P < 0.001).

Based on our finding that miR-489-3p is lowly expressed in PC, we speculated that miR-489-3p could inhibit the progression of pancreatic cancer. Therefore, we transfected PC cells with miR-NC, miR-489-3p mimics, anti-miR-NC and anti-miR-489-3p inhibitors (**Figure 2A**). The CCK8 and plate cloning experiments showed that overexpression of miR-489-3p inhibited PC cell viability and clone formation ability, while the inhibition of miR-489-3p increased the PC cell growth ability (**Figures 2B–D**). The transwell experiment revealed that overexpression of miR-489-3p could inhibit the migration and invasion ability of PC cells (**Figures 2E, F**).

### MiR-489-3p Targets LDHA and PKM2

**Figure 3:**
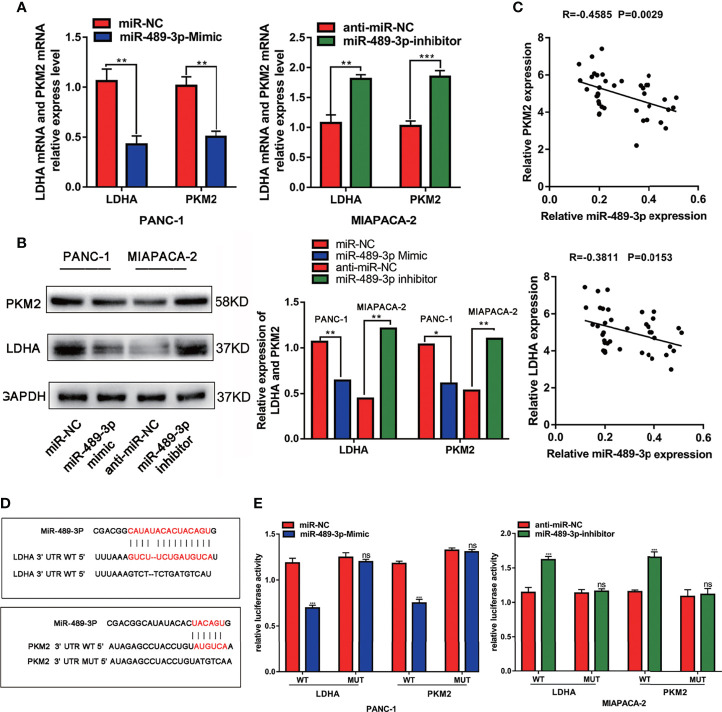
MiR-489-3p targets LDHA and PKM2. **(A, B)** qRT-PCR and western blot assays showed relative expression of LDHA and PKM2 after PC cells transfected with miR-489-3p mimics and miR-489-3p inhibitor. **(C)** Spearman rank correlation analysis showed correlation between miR-489-3p and LDHA and PKM. **(D)** The predicted binding site of miR-489-3p in human LDHA and PKM gene 3’ UTR, and the corresponding sequence in the mutated type. **(E)** Luciferase reporter gene assay analysis of the relationship between miR-489-3p and LDHA and PKM. (*P < 0.05, **P < 0.01, ***P < 0.001, ns, no significant).

Furthermore, we explored the mechanisms by which miR-489-3p regulate PC growth. We found that LDHA and PKM2 were targets of miR-489-3p by STARBASE3.0 (http://starbase.sysu.edu.cn/) (**Figures S1A, B**). Therefore, we supposed that miR-489-3p could be performing its biological function by targeting them. To verify this, we overexpressed and inhibited miR-489-3p in PC cells and found that LDHA and PKM2 were negatively regulated by miR-49-3p (**Figures 3A, B**). And the tissue co-expression correlation analysis indicated that miR-489-3p was negatively correlated with LDHA and PKM2 (**Figure 3C**). The binding sequences and mutation sites of LDHA and PKM2 on miR-489-3p are shown in **Figure 3D**. Overexpression of miR-489-3p significantly reduced the luciferase activity of wild types (WT) of LDHA and PKM2 but not their mutants (MUT). In contrast, down-regulating miR-489-3p significantly increased the luciferase activity of WT of LDHA and PKM2 but did not have any effect on their mutation (**Figure 3E**). These results indicate that miR-489-3p could target LDHA and PKM2.

### MiR-489-3p Regulates PC Glycolysis

**Figure 4:**
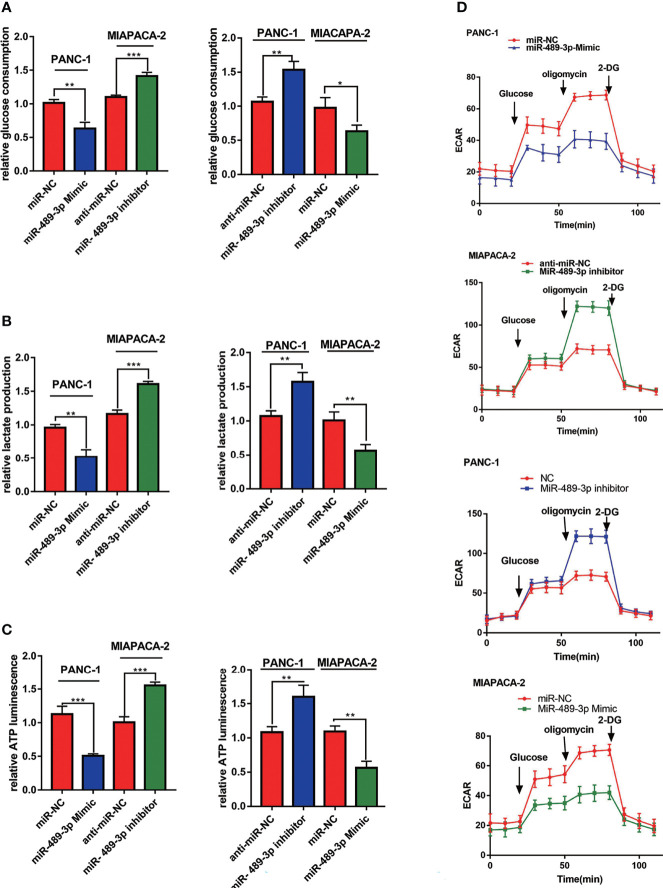
MiR-489-3p regulates PC glycolysis. **(A–C)** Cell metabolism experiments showed glucose uptake, lactic acid production, and ATP production of miR-489-3p mimics and miR-489-3p inhibitors in PC cells. **(D)** The hippocampal XF extracellular flux analyzer analysis of the PC ECAR in miR-489-3p mimics and inhibitors group. (*P < 0.05, **P < 0.01, ***P < 0.001).

Since miR-489-3p can target LDHA and PKM2 which were glycolysis related enzymes, we hypothesized that miR-489-3p could regulate glycolysis. Therefore, we performed glycolysis related experiments to research the metabolic of PC tumor cells after overexpression or inhibition of miR-489-3p. We noticed a reduction in the production of lactate, glucose, and ATP in the PC cells after miR-489-3p transfected with miR-489-3p mimic. Conversely, after the downregulation of miR-489-3p, the production of all these compounds increased (**Figures 4A**–**C**). Then, we found that reducing the expression of miR-489-3p inhibited the extracellular acidification rate (ECAR) of PC cells (**Figure 4D**).

### LDHA and PKM2 Restored miR-489-3p Mediated Proliferation and Invasion in PC

**Figure 5:**
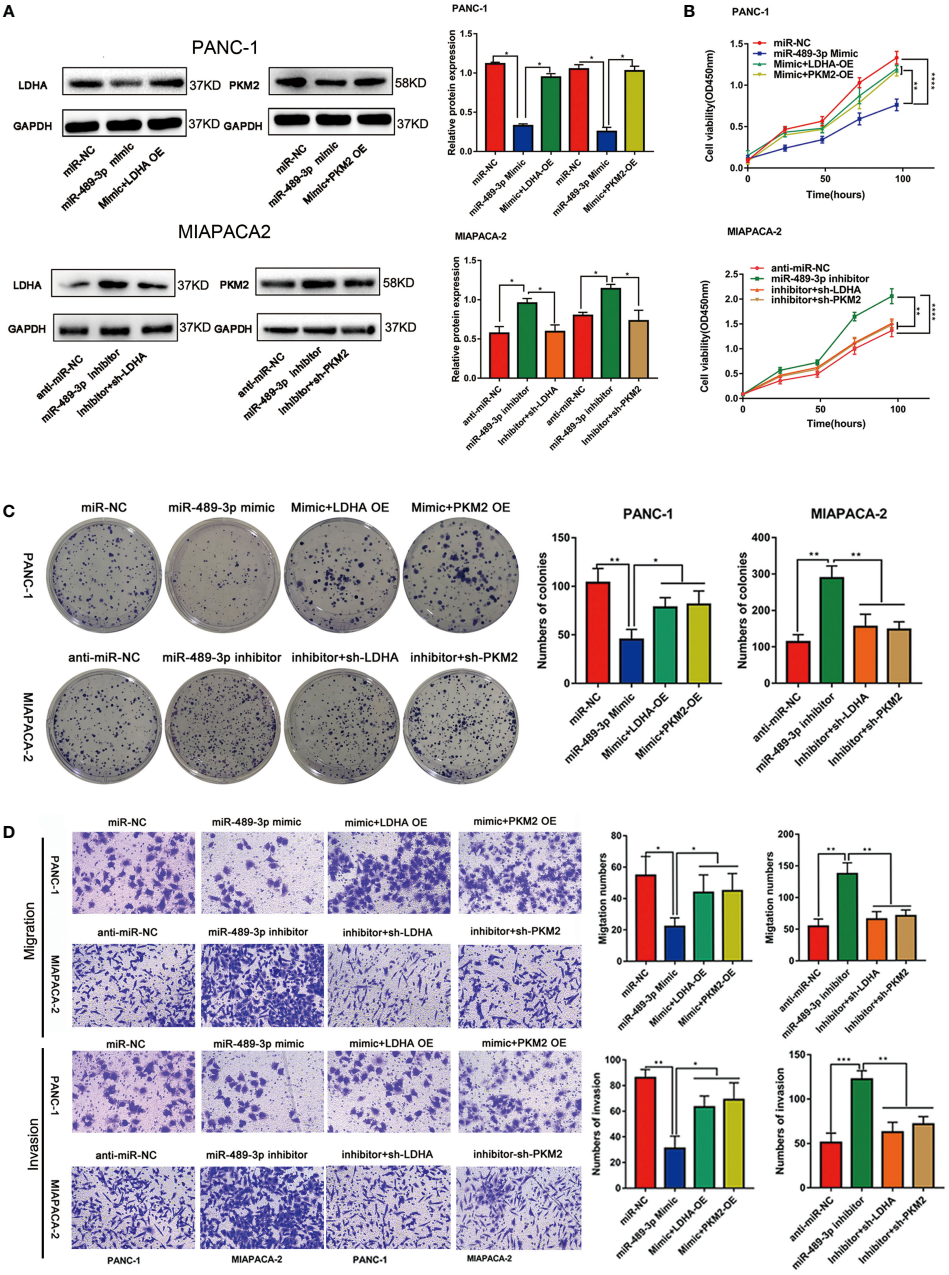
LDHA and PKM2 restored the function of miRNA-mediated proliferation and metastasis ability. **(A)** Western blot experiments showed that the relative expression of LDHA and PKM2 in PC cells transfected with miR-489-3p mimic, inhibitor, LDHA, PKM2 overexpressed plasmid or shRNA. **(B, C)** CCK8 and plate cloning and transwell migration assays showed that the proliferation ability of PC cells transfected with miR-489-3p mimic, inhibitor, LDHA, PKM2 overexpressed plasmid or shRNA. **(D)** Transwell assays show that the migration and invasion ability of PC cells transfected with miR-489-3p mimic, inhibitor, LDHA, PKM2 overexpressed plasmid or shRNA. (*P < 0.05, **P < 0.01, ***P < 0.001, ****P < 0.0001).

Our previous results demonstrated that miR-489-3p could regulate the progression of pancreatic cancer and target LDHA and PKM2. Therefore, we hypothesized that miR-489-3p might be carrying out its function by targeting LDHA and PKM2. The qRT-PCR and western blot assays showed that overexpression of miR-489-3p could down-regulate LDHA and PKM2, and overexpression of LDHA and PKM2 could restore this effect. Similarly, miR-489-3p inhibitor up-regulated LDHA and PKM2, and down-regulating LDHA and PKM2 restored this phenomenon (**Figures S2A**, **5A**). Then, CCK8 and plate cloning assays showed that miR-489-3p could target LDHA and PKM2 to regulate the proliferation of pancreatic cancer cells (**Figures 5B, C**, **S2B, C**). Transwell assays showed that miR-489-3p could regulate the migration and invasion ability of PC cells through LDHA and PKM2 (**Figures 5D**, **S2D**).

### MiR-489-3p Suppresses Glycolysis Through LDHA and PKM2

**Figure 6:**
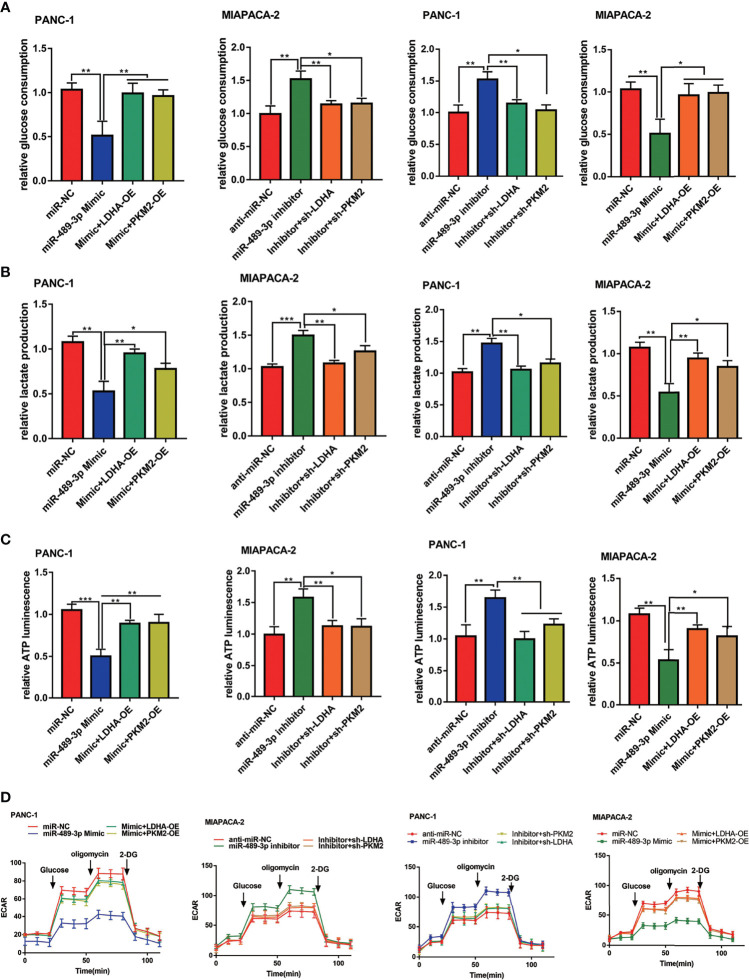
MiR-489-3p suppresses glycolysis through LDHA and PKM2. **(A–C)** Cell metabolism experiments showed that glucose uptake, lactic acid production, and ATP production of PC cells transfected with miR-489-3p mimic, inhibitor, LDHA, PKM2 overexpressed plasmid or shRNA. **(D)** The hippocampal XF extracellular flux analyzer analysis of the PC ECAR after transfected with miR-489-3p mimic, inhibitor, LDHA, PKM2 overexpressed plasmid or shRNA. (*P < 0.05, **P < 0.01, ***P < 0.001).

To confirm whether miR-489-3p also inhibited glycolysis of PC cells by targeting LDHA and PKM2, we overexpressed and knocked down of LDHA and PKM2 in the miR-489-3p mimic or inhibitor group PC cells. miR-489-3p significantly reduced glucose consumption, lactic acid production, and ATP production, whereas the effect was reversed by LDHA or PKM2 overexpressed. Likewise, down-regulating miR-489-3p markedly increased PC cells glycolysis, whereas LDHA or PKM2 downregulation reduced the glycolysis (**Figures 6A**–**C**). In addition, the hippocampal XF extracellular flux analyzer showed that LDHA and PKM2 could restore the extracellular acidification rate (ECAR) caused by miR-489-3p (**Figure 6D**).

### Effects of Overexpression of miR-489-3P on PC Proliferation and Metabolism *In Vivo*

**Figure 7:**
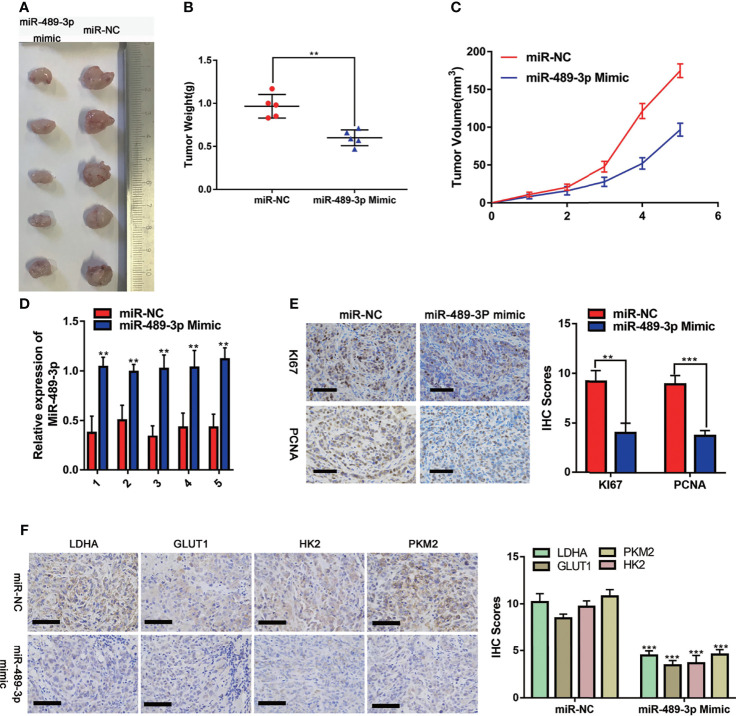
Effects of overexpression of miR-489-3P on PC proliferation and metabolism *in vivo*. **(A)** Typical images of nude mice tumors (n = 5), **(B)** subcutaneous tumor weight, **(C)** subcutaneous tumor volume, **(D)** miR-489-3p expression in xenografts by qRT-PCR. **(E)** Typical IHC staining images of xenografts show Ki-67 and PCNA expression. The bar stands for 50 microns. **(F)** Typical IHC staining images of xenografts show the expression of metabolic indicators (LDHA, GLUT1, HK2, PKM2). The bar stands for 100 microns. (**P < 0.01, ***P < 0.001).

To investigate the effect of miR-489-3p on tumor progression *in vivo*, we used a xenograft tumor model. The image (**Figure 7A**) was taken after 35 days of subcutaneous tumor formation in nude mice. Changes in tumor weight and volume indicated that overexpression of miR-489-3p could inhibit tumor growth (**Figures 7B, C**). The expression of miR-489-3p was significantly lower in the control group than in the samples transfected with miR-489-3p mimics (**Figure 7D**). The IHC assay indicated that cell proliferation factors KI-67 and PCNA were downregulated in miR-489-3p overexpression group (**Figure 7E**). Finally, to investigate whether miR-489-3p regulating glycolysis, the IHC results showed that tumor glycolysis markers was significantly inhibited after miR-489-3p overexpressed (**Figure 7F**).

## Discussion

Pancreatic cancer is a highly malignant tumor of the digestive tract, which is extremely difficult to diagnose and treat and accounts for 8% of cancer-related mortality worldwide (23). The highly invasive ability is one of the causes of the lethality of pancreatic cancer. The occurrence and development of tumors are related to the abnormal expression of specific genes (24). Therefore, identifying genes related to the growth of pancreatic cancer is very important in the research and treatment of this disease. Scientists have long found that glycolysis intensity is positively correlated with tumor invasion and metastasis (25, 26). For example, lactic acid, the product of glycolysis, forms an acidic microenvironment that is essential for the transformation of lung metastases to malignant metastases (25). The inhibition of glycolysis to limit the energy supply to the cancer cells has become an emerging chemotherapy approach.

The miR-489-3p is known to inhibit the growth of a variety of tumors, such as bladder cancer (27), renal cell carcinoma (28), and osteosarcoma (18). However, its relationship with pancreatic cancer has not been well established. Based on the tissue microarray analysis, the present study found that the expression of miR-489-3p in pancreatic cancer was lower than that in adjacent tissues, and it was closely related to the prognosis of clinical patients. Our functional experiments also demonstrated that miR-489-3p could inhibit the proliferation and metastasis of pancreatic cancer cells. However, the mechanism by which overexpression of miR-489-3p inhibits pancreatic cancer growth metabolism is not yet clear.

Furthermore, a luciferase reporter experiment showed that miR-489-3p could target the expression of LDHA and PKM2. LDHA catalyzes the conversion of pyruvate to lactic acid during glycolysis whereas PKM2 is a rate-limiting enzyme for glycolysis (29). Previous studies have shown that LDHA is inseparable from the aerobic glycolysis of tumors (30). There are two isomers of M-type pyruvate kinase: PKM1, PKM2 and PKM2 is the only form of pyruvate kinase found in cancerous tissues (31, 32). A transcription factor Hypoxia-Inducible Factor (HIF) is highly expressed in tumors, and one of its subtypes, HIF-1, promotes the expression of the promoter region of the GLUT1, HK2, MYC gene (33). GLUT1, HK2 are the vital glycolysis enzymes regulating the glucose metabolic in cancer cell (34). MYC is a transcription factor with a wide range of biological functions, including cellular energy metabolism (35). It can stimulate the expression of many genes, including LDHA and PKM2 (36, 37). Since LDHA and PKM2, the targets of miR-489-3p are glycolysis-related enzymes, we confirmed that miR-489-3p could inhibit glycolysis of pancreatic cancer cells.

We then performed functional and glycolysis-related experiments by overexpression or knockdown of LDHA or PKM2. Apparently, the overexpression of LDHA or PKM2 restored the inhibition of pancreatic cancer cell growth and glycolysis caused by overexpression of miR-489-3p. In contrast, knocking down LDHA or PKM2 alleviated the fast growth and hypermetabolism of pancreatic cancer cells caused by inhibition of miR-489-3p. On performing the *in vivo* experiments, we found that the xenograft tumors in the overexpressing miR-489-3p group were significantly smaller than the NC group. Besides, the immunohistochemistry of the xenograft tumors showed that the proliferative and metabolic markers of the overexpressing miR-489-3p group were lower than the NC group.

In recent years, studies have found that the analysis of circulating miRNAs may improve the choice of the best treatment strategy (38). At present, some pre-clinical and clinical trials have used miRNA as a target for the early diagnosis and treatment of tumors including pancreatic cancer (39). However, due to a series of difficulties such as miRNA carrier, miRNA toxicity evaluation, and effectiveness testing, it cannot be used in clinical practice and treatment at present (40). And metabolic reprogram is necessary for successful metastasis and effective colonization of distant sites (41). Though it has been demonstrated that micro RNAs can control tumor growth by regulating glycolysis-related genes (42, 43). Our research demonstrated that aberrant expression of miR-489-3p could modulate the progression of pancreatic cancer *via* targeting LDHA and PKM2. It may expand the biomarker library for pancreatic cancer. In summary, our *in vivo* and *in vitro* experiments have demonstrated that miR-489-3p inhibits the growth and glycolysis of pancreatic cancer cells. This study has provided new ideas for molecular targeted therapy of pancreatic cancer.

## Data Availability Statement

The original contributions presented in the study are included in the article/**Supplementary Material**. Further inquiries can be directed to the corresponding author.

## Ethics Statement

The studies involving human participants were reviewed and approved by the Institutional Ethics Committee of Renmin Hospital of Wuhan University. The patients/participants provided their written informed consent to participate in this study. The animal study was reviewed and approved by the Institutional Ethics Committee of Renmin Hospital of Wuhan University.

## Author Contributions

JJ and ZH contributed to the experiment design, and data analysis. DZ, YS, and JW contributed to the experiment implementation. TL and JJ contributed to manuscript draft and data analysis. All authors read and approved the final manuscript.

## Funding

This work was supported by a National Natural Science Foundation of China (NSFC) grant (No. 81572429 awarded to JJ).

## Conflict of Interest

The authors declare that the research was conducted in the absence of any commercial or financial relationships that could be construed as a potential conflict of interest.

## Publisher’s Note

All claims expressed in this article are solely those of the authors and do not necessarily represent those of their affiliated organizations, or those of the publisher, the editors and the reviewers. Any product that may be evaluated in this article, or claim that may be made by its manufacturer, is not guaranteed or endorsed by the publisher.

## Supplementary Material

Supplementary Figure 1
**(A, B)** Bioinformation analysis the miRNA and targets *via* the prediction database of STARBASE.

Supplementary Figure 2 LDHA and PKM2 restored the function of miRNA-mediated proliferation and metastasis ability. **(A)** q-RT-PCR showed that the relative expression of LDHA and PKM2 in PC cells transfected with miR-489-3p mimic, inhibitor, LDHA, PKM2 overexpressed plasmid or shRNA. **(B, C)** CCK8 and plate cloning and transwell migration assays showed that the proliferation ability of PC cells transfected with miR-489-3p inhibitor, LDHA, PKM2 shRNA. **(D)** Transwell assays show that the migration and invasion ability of PC cells transfected with miR-489-3p inhibitor, LDHA, PKM2 shRNA. (*P < 0.05, **P < 0.01, ***P,< 0.001, ****P<0.0001).
