# Supplementary figures and images for: MiR-489-3p Reduced Pancreatic Cancer Proliferation and Metastasis By Targeting PKM2 and LDHA Involving Glycolysis
Source: Front Oncol. 2021 Nov 12;11:651535. doi: 10.3389/fonc.2021.651535 (PMC8632778; doi:10.3389/fonc.2021.651535)

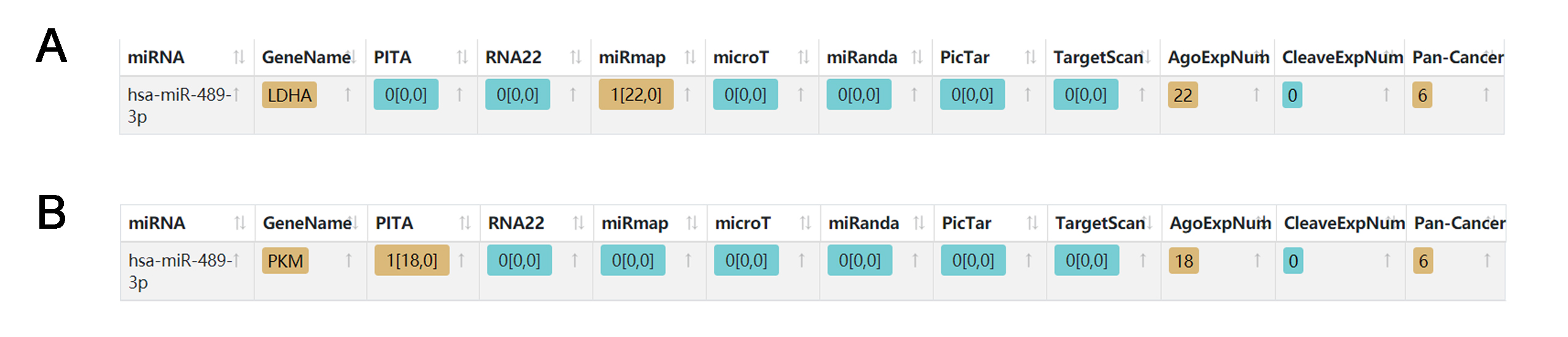

Supplement: Supplementary Figure 1 — (A, B) Bioinformation analysis the miRNA and targets via the prediction database of STARBASE. [file Image_1.jpeg]

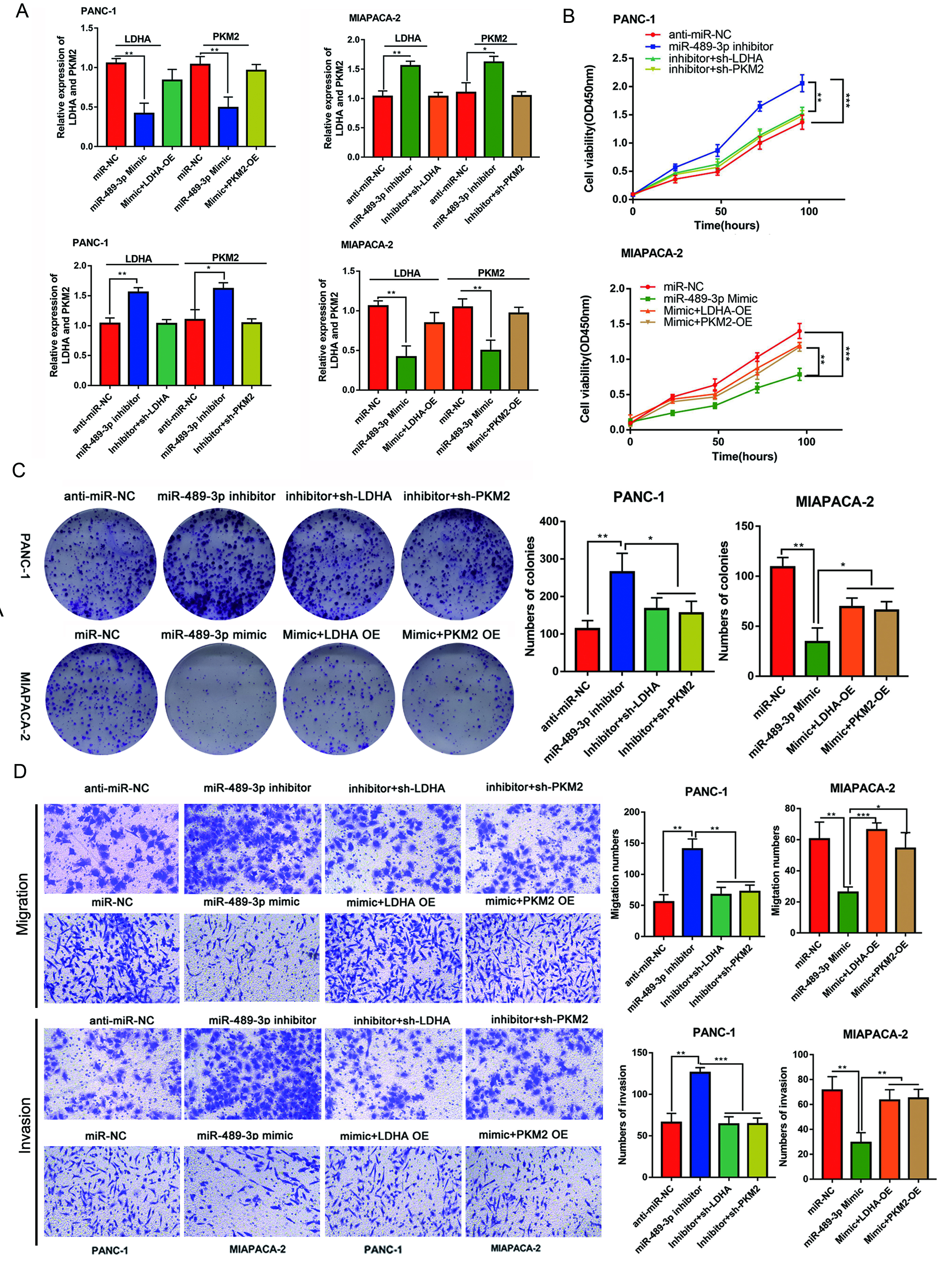

Supplement: Supplementary Figure 2 — LDHA and PKM2 restored the function of miRNA-mediated proliferation and metastasis ability. (A) q-RT-PCR showed that the relative expression of LDHA and PKM2 in PC cells transfected with miR-489-3p mimic, inhibitor, LDHA, PKM2 overexpressed plasmid or shRNA. (B, C) CCK8 and plate cloning and transwell migration assays showed that the proliferation ability of PC cells transfected with miR-489-3p inhibitor, LDHA, PKM2 shRNA. (D) Transwell assays show that the migration and invasion ability of PC cells transfected with miR-489-3p inhibitor, LDHA, PKM2 shRNA. (*P < 0.05, **P < 0.01, ***P,< 0.001, ****P<0.0001). [file Image_2.jpg]
